# Taurine suppresses ROS-dependent autophagy via activating Akt/mTOR signaling pathway in calcium oxalate crystals-induced renal tubular epithelial cell injury

**DOI:** 10.18632/aging.103730

**Published:** 2020-09-15

**Authors:** Yan Sun, Shiting Dai, Jin Tao, Yunlong Li, Ziqi He, Quan Liu, Jiawen Zhao, Yaoliang Deng, Juening Kang, Xuepei Zhang, Sixing Yang, Yunlong Liu

**Affiliations:** 1 Department of Urology, The First Affiliated Hospital of Zhengzhou University, Zhengzhou, China; 2 Department of Urology, Renmin Hospital of Wuhan University, Wuhan, China; 3 Department of Urology, The First Affiliated Hospital of Guangxi Medical University, Nanning, China

**Keywords:** autophagy, calcium oxalate crystals, reactive oxygen species, renal tubular epithelial cells, taurine

## Abstract

Oxidative stress and autophagy are the key promoters of calcium oxalate (CaOx) nephrolithiasis. Taurine is an antioxidant that plays a protective role in the pathogenesis of kidney disease. Previous studies found that taurine suppressed cellular oxidative stress, and inhibited autophagy activation. However, the effect of taurine on CaOx kidney stone formation remains unknown. In the present work, we explored the regulatory effects of taurine on CaOx crystals-induced HK-2 cell injury. Results showed that pretreatment with taurine significantly enhanced the viability of HK-2 cells and ameliorated kidney tissue injury induced by CaOx crystals. Taurine also markedly reduced the levels of inflammatory cytokines, apoptosis, and CaOx crystals deposition. Furthermore, we observed that taurine supplementation alleviated CaOx crystals-induced autophagy. Mechanism studies showed that taurine reduced oxidative stress via increasing SOD activity, reducing MDA concentration, alleviating mitochondrial oxidative injury, and decreasing the production of intracellular ROS. Taurine treatment also effectively activated Akt/mTOR signaling pathway in CaOx crystals-induced HK-2 cells both *in vitro* and *in vivo*. In summary, the current study shows that taurine inhibits ROS-dependent autophagy via activating Akt/mTOR signaling pathway in CaOx crystals-induced HK-2 cell and kidney injury, suggesting that taurine may serve as an effective therapeutic agent for the treatment of CaOx nephrolithiasis.

## INTRODUCTION

Nephrolithiasis is a commonly diagnosed disease worldwide with an incidence rate of 10-15%. Despite the advances in minimally invasive surgical techniques, the recurrence rate of kidney stones is still around 50% within 5-10 years after the initial onset [1]. Thus, there is an urgent need to find a therapeutic agent with satisfactory therapeutic effects and small side effects for nephrolithiasis patients.

Calcium oxalate (CaOx) stone is the most common component of urinary stones that accounts for 80% of all cases [2]. The formation of CaOx stone is a complex process and the underlying mechanism remains unclear. Renal tubular epithelial cell (RTEC) injury plays a key role in kidney stone formation. The renal tubular epithelial damage may facilitate the transfer of crystals from renal tubules to renal interstitium, contributing to the development of Randall's plaques [3]. Randall's plaque at the surface of renal papilla results from inflammatory responses. Therefore, RTEC injury is a critical predisposing factor for the aggregation, nucleation, growth, and deposition of CaOx crystals, and eventually the formation of kidney stones [4]. RTEC damage is also closely related to reactive oxygen species (ROS)-induced oxidative stress [5]. Drugs suppressing the levels of ROS and oxidative stress in renal cells might be used for the treatment of kidney stones.

ROS acts as an important signaling molecule in the pathogenesis of various diseases. Taurine (Tau) is an important antioxidant that shows protective effects on kidneys [6, 7]. It has been reported that Tau increases the activities of SOD and GSH-Px in kidney tissues and alleviated ethylene glycol-induced oxidative injury in a rat nephrolithiasis model. However, the specific molecular mechanisms have not been revealed [8].

Previous evidence showed that the level of autophagy in patients with CaOx kidney stones was significantly higher than that in healthy controls and CaOx crystals promoted the production of ROS in RTEC, which mediated the activation of autophagy [9, 10]. On the contrary, the inhibition of autophagy effectively attenuated CaOx crystals-induced oxidative stress damage, mitochondrial injury, urinary oxalate excretion, renal crystal deposition, and finally inhibited the formation of CaOx crystals in rat kidneys [9–11]. Thus, ROS-mediated autophagy may play an important role in RTEC damage caused by kidney stones.

A recent study on rat offspring showed that Tau reduced the production of ROS in the pancreas tissues exposed to arsenic trioxide via activating Nrf2 and Trx, and also alleviated autophagic damage in the pancreas [12]. Kaneko et al. demonstrated that Tau suppressed the activation of ERK signaling pathway by decreasing ROS generation, thereby accelerating autophagic flux [13]. These data suggested that Tau inhibited the production of intracellular ROS induced by different stimuli, thereby inhibiting or promoting autophagic cell death. However, the regulation of Tau in CaOx crystals-induced RTEC injury is still unclear. In this study, we aimed to investigate whether Tau inhibited CaOx crystals-induced RTEC injury by suppressing ROS-dependent autophagy. These results demonstrated the pharmacological properties of Tau in the formation of CaOx kidney stones and supported its potential use in the management of nephrolithiasis.

## RESULTS

### Tau attenuates CaOx crystals-induced cell injury

**Figure 1:**
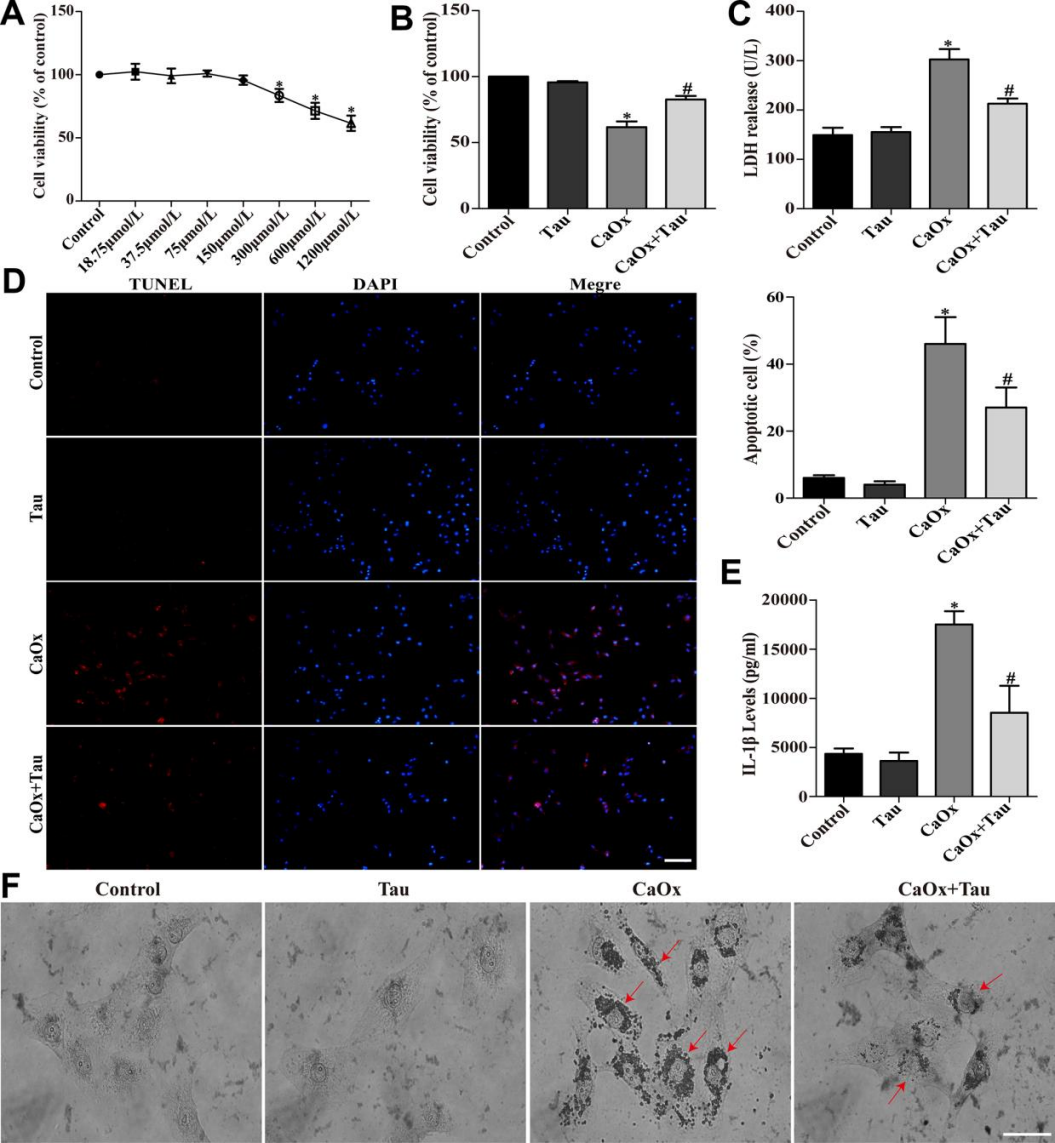
**Tau ameliorated CaOx crystals-induced HK-2 cell injury.** (**A**) Effect of Tau on the viability of HK-2 cells. (**B**) Effect of Tau on the viability of cells exposed to CaOx crystals. (**C**) The concentration of secreted LDH in cell supernatants. (**D**) The apoptosis of HK-2 cells was assessed using TUNEL assay; scale bar: 50 μm. (**E**) ELISA detection of IL-1β expression in culture supernatants. (**F**) Observation of the crystal adhesion on HK-2 cells under a light microscope. Red arrows indicate intracellular CaOx crystals; scale bar: 20 μm. Data are presented as the mean ± SD (n=3). ^*^P < 0.05 versus the control group, ^#^*P* < 0.05 versus the CaOx group.

Tau at 0 to 150 μmol/L showed no obvious cytotoxicity on HK-2 cells (Figure 1A). Therefore, a dose of 150 μmol/L was selected for further experiments to avoid the interference of drug toxicity on the experiment. CaOx crystal stimulation significantly decreased cell viability as compared to the control groups. Pretreatment with 150 μmol/L Tau significantly improved cell viability (Figure 1B). The secretion of LDH (Figure 1C) and IL-1β (Figure 1E) induced by CaOx crystals and crystal adhesion (Figure 1F) was significantly decreased by the treatment with Tau. The increased apoptosis following CaOx stimulation was also decreased by Tau pretreatment (Figure 1D).

### Tau ameliorates EG-induced renal injury and CaOx crystal deposition in rat kidneys

**Figure 2:**
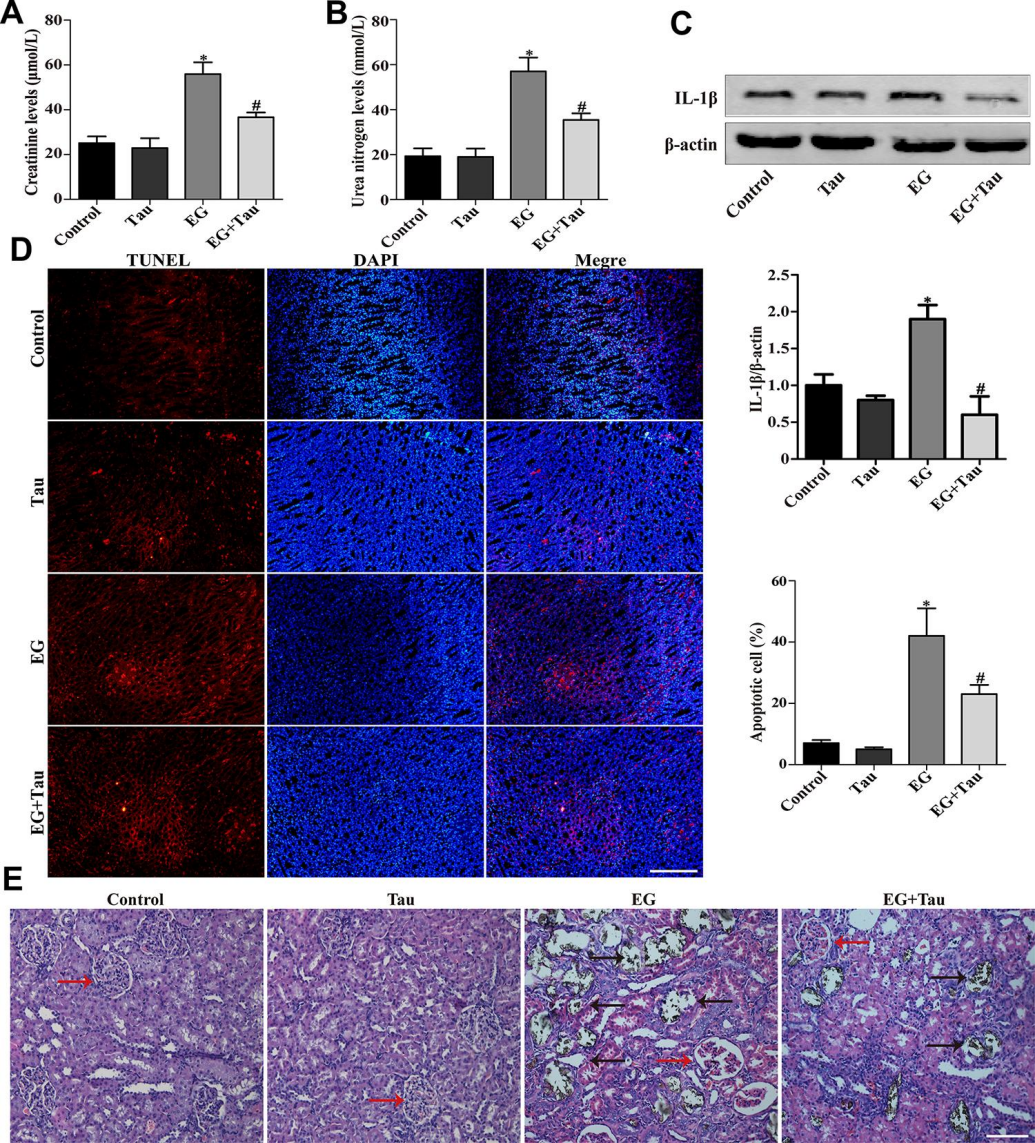
**Tau attenuates EG-induced renal damage and crystal deposition in rat kidneys.** (**A**) Effect of Tau on the serum expression of creatinine after EG-induced renal injury. (**B**) Effect of Tau on urea nitrogen following EG-induced renal injury. (**C**) Representative immunoblot and quantification analysis of IL-1β expression. (**D**) Renal tissue apoptosis was assessed by TUNEL staining; scale bar: 200 μm. (**E**) Kidney injury and crystal deposition were determined using Von Kossa-staining. Red and black arrows indicate glomerulus and crystal deposition, respectively; scale bar: 200 μm. Data are presented as the mean ± SD (n=3).^*^P < 0.05 versus the control group, ^#^*P* < 0.05 versus the EG group.

As shown in Figure 2A and Figure 2B, the elevated serum levels of creatinine and urea nitrogen in EG group were attenuated in EG + Tau group. The treatment with Tau also reduced EG-induced renal damage, as evidenced by a significant reduction in IL-1β expression (Figure 2C). Then we examined the crystal deposition in rat renal tubules by Von Kossa staining and found that EG led to crystal retention in rat kidneys. EG group exhibited histopathological alterations and significantly increased crystal deposition in the kidney compared to the controls, indicating the damage to the tubules and glomeruli. Treatment with Tau significantly decreased EG-induced kidney damage and renal crystal deposition (Figure 2E). Moreover, we detected apoptosis in rat kidney tissues using the TUNEL assay kit and found that EG-induced renal crystal deposition is accompanied by an increase in apoptosis, which was significantly reduced by Tau treatment (Figure 2D).

### Tau inhibits CaOx crystals-induced autophagic process in cells and rat kidneys

**Figure 3:**
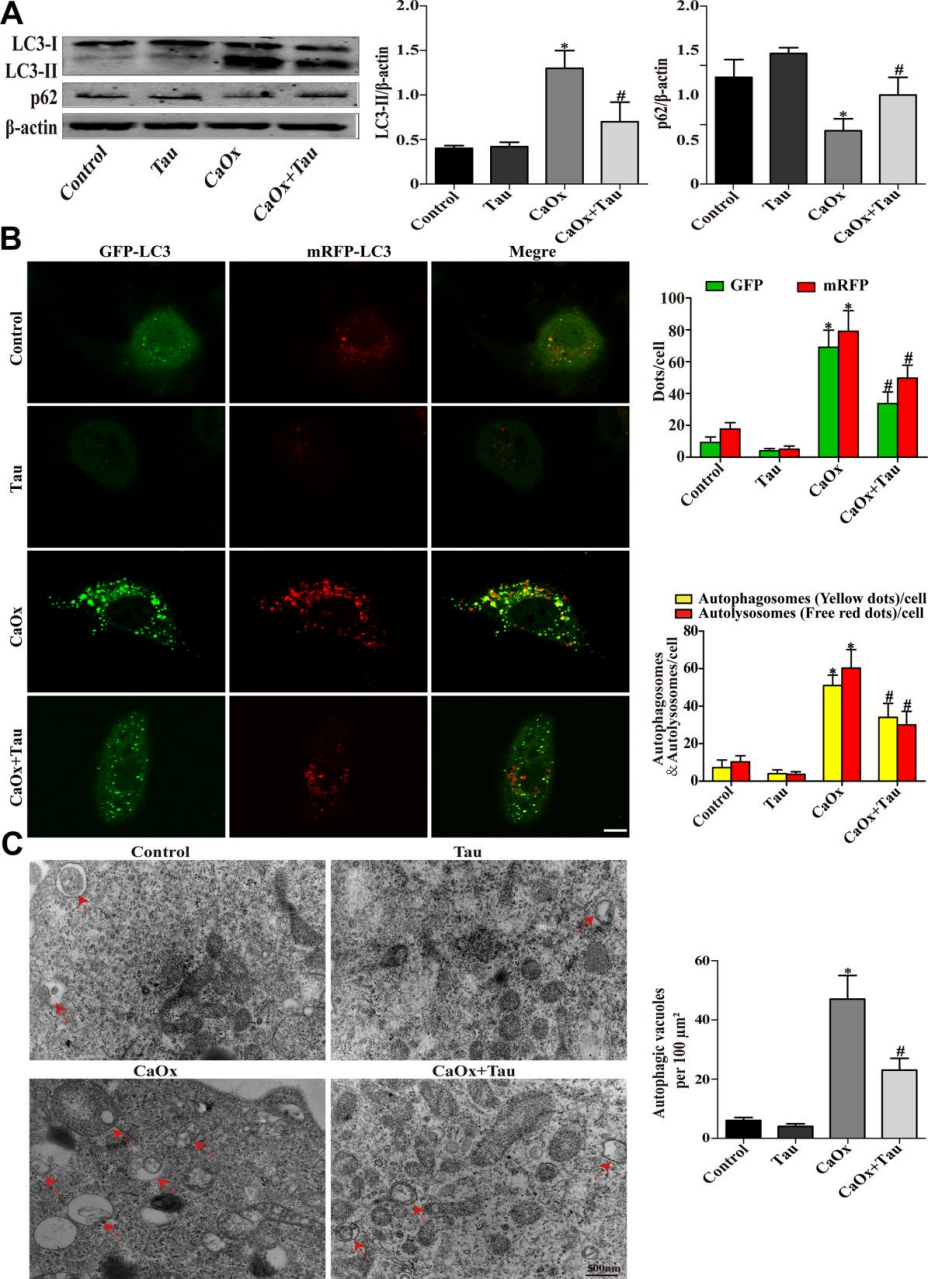
**Effects of Tau on CaOx crystals-induced autophagy in cells.** (**A**) The expressions of LC3-II and p62 were assessed by Western blot. (**B**) Fluorescence microscopy and quantitative analysis of cells transduced with Ad-mRFP-GFP-LC3. The green GFP dots and the red mRFP dots were used to label and track LC3. In the merged image, the yellow dots and the red dots indicate autophagosomes and autolysosomes, respectively; scale bar: 50 μm. (**C**) Detection of autophagic vacuoles by TEM in HK-2 cells. Red arrows: autophagic vacuoles; scale bar: 500 nm. Data are presented as the mean ± SD (n=3). ^*^P < 0.05 versus the control group, ^#^*P* < 0.05 versus the CaOx group.

**Figure 4:**
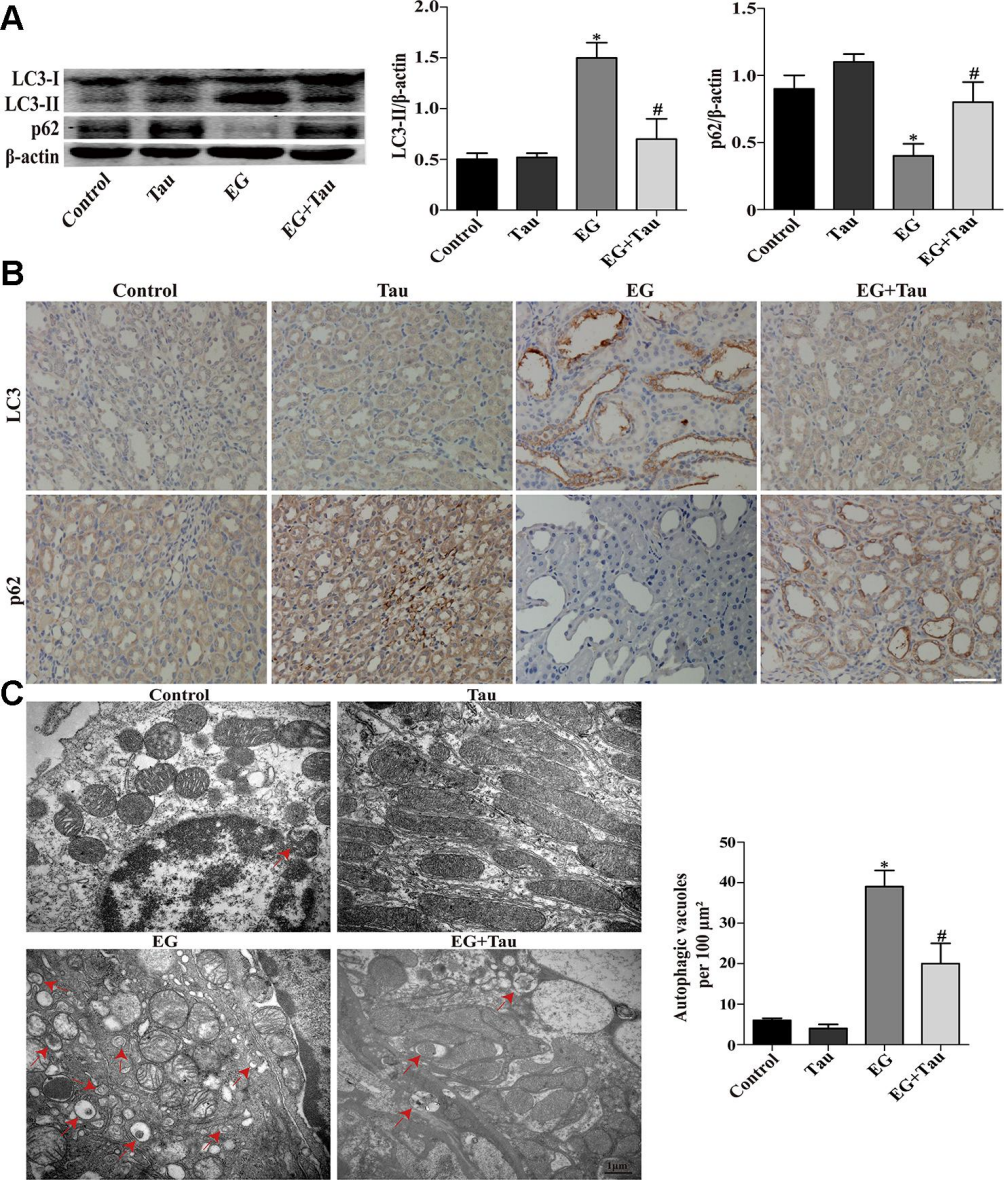
**Effects of Tau on EG-induced autophagic activity in rat kidneys.** (**A**) The levels of LC3-II and p62 were examined by Western blot. (**B**) The expressions of LC3 and p62 in kidney tissues were detected by immunohistochemical staining; scale bar: 100 μm. (**C**) Detection of autophagic vacuoles in renal tissues by TEM. Red arrows: autophagic vacuoles; scale bar: 1 μm. Data are presented as the mean ± SD (n=3). ^*^P < 0.05 versus the control group, ^#^*P* < 0.05 versus the EG group.

During the process of autophagy, cytosolic LC3 (LC3-I) enzymatically removes a small segment of polypeptide and transforms into autophagosomes (LC3-II). The increase in LC3-II represents the initiation of autophagy. P62 is a protein complex that can be degraded by autophagy. The elevation of LC3-II and the simultaneous decrease in P62 indicate that autophagy is activated. Here, the expression of the key autophagy protein LC3B (LC3-II / LC3-I) in the model group was significantly upregulated compared to the controls. The model group also presented significantly reduced P62 expression and an increased number of autophagic vesicles. Treatment with Tau significantly reduced the excessive activation of autophagy (Figure 3A and Figure 4A) and the formation of autophagic vesicles (Figure 3C and Figure 4C). Immunohistochemistry results were consistent with those of Western blot (Figure 4B). We further transduced HK-2 cells with double-labeled adenovirus to detect autophagic flux. Autolysosomes and autophagosomes were marked as red and yellow dots, respectively, in the merged images. CaOx stimulation significantly increased the green, red, and yellow spots, whereas Tau pretreatment significantly suppressed the formation of autophagic vesicles (Figure 3B).

### Tau downregulates autophagy activation by inhibiting CaOx crystals-induced oxidative stress both *in vitro* and *in vivo*

**Figure 5:**
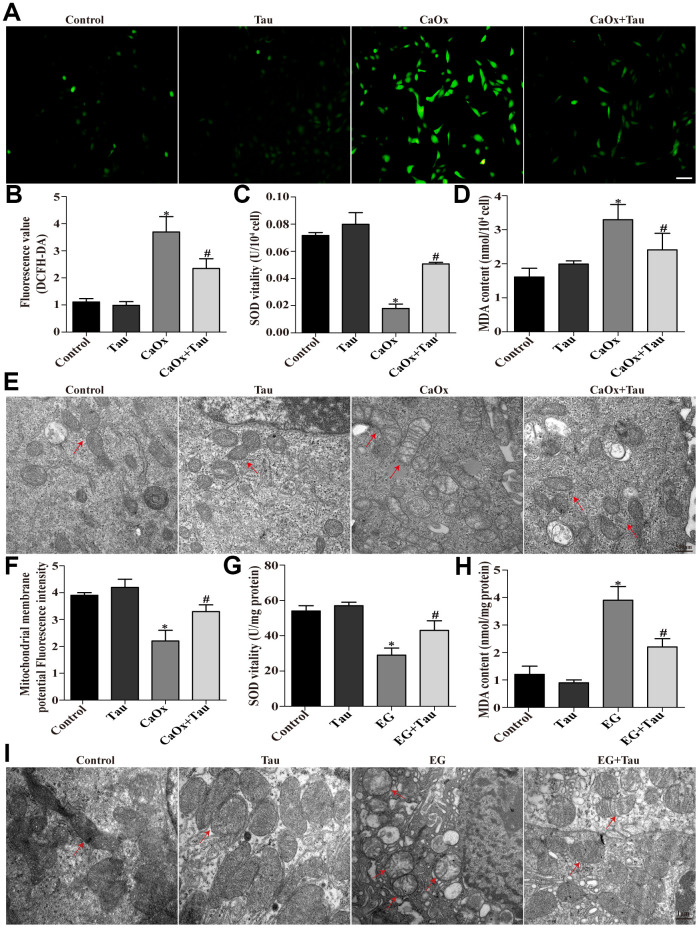
**Tau alleviates oxidative injury induced by CaOx crystals.** (**A**) The ROS level in HK-2 cells after different treatments was examined using DCFH-DA; scale bar: 50 μm. (**B**) Quantitative analyses of DCFH-DA assay. (**C** and **D**) Effects of Tau on SOD and MDA levels in cells treated with CaOx crystals. (**E**) The ultrastructural morphology of the was detected by TEM. Red arrows: mitochondria; scale bar: 500 nm. (**F**) Mitochondrial membrane potential of all groups of cells. (**G** and **H**) Effects of Tau on SOD and MDA levels in EG-induced renal tissues. (**I**) The ultrastructural morphology of mitochondria in renal tissues was detected by TEM. Red arrows: mitochondria; scale bar: 1 μm. Data are presented as the mean±SD (n=3). ^*^P < 0.05 versus the control group, ^#^*P* < 0.05 versus the CaOx group or the EG group.

Mitochondrion is one of the most sensitive organelles that can be damaged by excessive oxidation. It has been recognized as the main source of intracellular ROS. We first assessed the damage of HK-2 cells and rat kidneys by using transmission electron microscopy. The mitochondria in Tau group were basically normal compared to the controls, while swelling and deformed mitochondria were found in the model group. Treatment with Tau significantly alleviated mitochondrial damage caused by crystal stimulation (Figure 5E and 5I). Subsequently, we observed that pretreatment with Tau enhanced the mitochondrial membrane potential in cells induced by CaOx crystals (Figure 5F). Furthermore, via examining oxidative stress-related factors, we found that crystal stimulation significantly increased ROS production (Figure 5A and 5B) in HK-2 cells, which was accompanied by a reduction in SOD (Figure 5C) and an upreuglation in MDA (Figure 5D). The results of the animal model were consistent with that in cell culture (Figure 5G and 5H). In contrast, treatment with Tau exerted an opposite effect on CaOx crystals-induced oxidative stress.

### Tau suppresses the ROS-dependent autophagy via activating Akt/mTOR signaling

**Figure 6:**
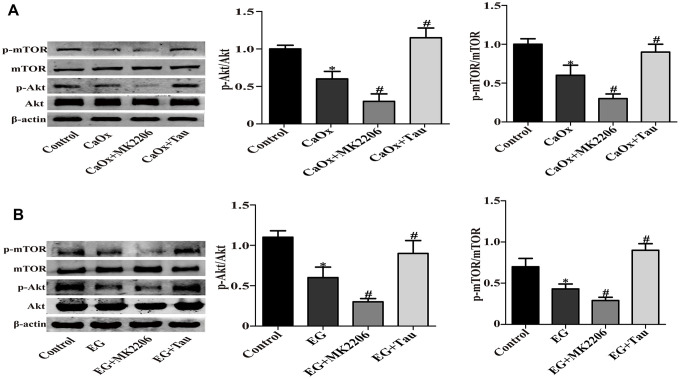
**Tau activates Akt/mTOR signaling pathway.** (**A**) The expressions of mTOR, P-mTOR, Akt, and P-Akt *in vitro*. (**B**) Representative immunoblot and quantification analysis of mTOR, Akt, P-mTOR, and P-Akt *in vivo*. Data are presented as the mean ± SD (n=3). ^*^P < 0.05 versus the control group, ^#^*P* < 0.05 versus the CaOx group or the EG group.

Akt/mTOR pathway is a classic pathway in the mediation of autophagy. We hypothesized that Akt/mTOR signaling was involved in Tau-mediated autophagy. The model group showed significantly reduced phosphorylation of Akt and mTOR compared to the controls. Akt inhibitor MK2206 treatment can effectively inhibit the activation of Akt / mTOR signaling pathway, and further reduce the phosphorylation of Akt and mTOR compared to CaOx / EG group. However, Tau treatment effectively reversed the inhibition of Akt/mTOR pathway induced by crystal stimulation (Figure 6A and 6B). These findings indicated the involvement of Akt/mTOR pathway in Tau-mediated autophagy.

## DISCUSSION

A growing number of publications suggest that Tau exerts a protective effect on the pathogenesis of kidney disease [6]. In the present work, we showed that Tau treatment attenuated CaOx crystals-induced RTEC injury both *in vitro* and *in vivo*. Taurine (a.k.a β-aminoacetic acid) is a sulfur-containing non-protein amino acid that is mainly distributed in kidney, heart, brain with the regulatory effects on osmotic pressure, cell protection, anti-oxidation, anti-inflammatory, membrane stability, ion transport, etc [14, 15]. Our results showed that pretreatment with Tau enhanced cell viability, decreased LDH activity and the levels of Cr and BUN, and ameliorated kidney tissue injury induced by CaOx crystal. It has been reported that Tau effectively interrupts ethanol-induced inflammatory cycle and ameliorates kidney damage [16]. Kim et al. demonstrated that Tau protected against doxorubicin-induced acute kidney injury by inhibiting the expressions of apoptosis-related proteins and inflammatory cytokines [17]. Here, we observed the anti-inflammatory and anti-apoptotic effects of Tau in CaOx crystals-induced HK-2 cells, which was supported by the findings that Tau pretreatment remarkably reduced the expression of inflammatory cytokines and the number of TUNEL-positive cells. Finally, we showed that Tau effectively reduced CaOx crystal adhesion and deposition in rat kidneys. These data clearly indicated the protection of Tau against CaOx crystals-induced HK-2 cell injury and renal damage.

Excessive autophagy plays a deleterious role in the development of CaOx nephrolithiasis [18, 19]. Previous data suggested the cytoprotective properties of Tau, which regulated the formation of autophagic vacuoles in various types of tissues under different pathological conditions [20, 21]. In our study, Tau supplementation alleviated CaOx crystals-induced autophagy in RTEC both *in vitro* and *in vivo*. The administration of Tau significantly decreased the amount of autophagic vacuoles in HK-2 cells exposed to CaOx crystals and the kidney tissues in EG-treated rats. The changes of autophagy-related proteins, such as LC3-II and p62, suggesting that CaOx-induced autophagy was reduced in cells pretreated with Tau. The numbers of autolysosomes and autophagosomes were also markedly decreased in taurine-treated cells, indicating an impairment of autophagic flux. Taken together, these findings suggested that Tau effectively suppressed excessive autophagy in RTEC in response to CaOx crystals.

Intriguingly, Rovetta et al. found that pre-conditioning with Tau increased the level of autophagy, decreased apoptosis and ER stress in NRK-52E cells induced by cisplatin [22]. On the contrary, Li et al. showed that Tau alleviated oxidative stress, ROS production, apoptosis occurrence, and excessive autophagy in DEHP-treated INS-1 cells [23]. Our results were consistent with the latter one, demonstrating that Tau exerted a protective effect on the apoptosis and inflammation of HK-2 cells through inhibiting CaOx-induced autophagic process. Whether Tau acts as a promoter or suppressor in the activation of autophagy may depend on different experimental conditions and stress stimuli.

Our previous study showed that ROS-mediated autophagy was involved in the formation of CaOx nephrolithiasis [9]. Accumulating evidence supports that taurine functions as an antioxidant to reduce ROS generation, inhibit cellular oxidative stress and autophagy activation [24, 25]. Our results showed that the administration of Tau distinctly decreased intracellular ROS production compared with untreated cells. SOD is the most important superoxide radical scavenging factor, whose activity indicates the capacity of cells against free radical damage [26]. MDA is the main product of lipid peroxidation *in vivo*, which reflects the level of lipid peroxidation and has been used as a key indicator for evaluating the degree of oxidative stress [27]. Our latest research showed that enhanced SOD activity inhibited autophagy-ERS response by reducing ROS production, and effectively alleviated ethylene glycol-induced renal damage and crystals deposition in a rat nephrolithiasis model [28]. Yang et al. reported that metformin significantly prevented the decrease of SOD and the increase of MDA, thereby attenuating oxidative stress damage induced by oxalate or hyperoxaluria [29]. Our current results were consistent with these studies, showing that treatment with Tau significantly decreased the level of cellular oxidative stress.

Mitochondria are the main source of ROS in the kidney [30]. Increasing evidence showed that Tau played a vital role in alleviating mitochondrial oxidative injury [31, 32]. In agreement with our previous data, this study showed that treatment with Tau effectively increased the level of mitochondrial membrane potential, attenuated mitochondrial edema and damage induced by CaOx crystals in HK-2 cells [8]. In addition, numerous reports have proven that mitochondrial ROS generation is involved in the regulation of autophagy activation [33, 34]. In summary, our data suggested that CaOx crystals activated autophagy through the oxidative stress-mitochondria-ROS axis, whereas Tau treatment protected RTEC via inhibiting this pathway.

PI3K/Akt/mTOR pathway plays a fundamental role in regulating cell growth, proliferation, and apoptosis [35]. mTOR is a highly conservative serine/threonine kinase that has been considered as an important regulator in the initial stage of autophagy [36]. Akt/mTOR signaling is associated with the upstream modulation of autophagy [37]. ROS activates autophagy through the inhibition of mTOR signaling [38]. A study reported that Tau blocked ROS-dependent autophagy by inhibiting AMPK/mTOR pathway, thereby suppressing OTA-promoted PCV2 replication [39]. Moreover, Li et al. found that Tau protected PC12 cells against methamphetamine-induced injury through decreasing ROS production and enhancing mTOR signaling, and eventually reducing autophagy [40]. These reports and our findings suggested that Tau inhibited CaOx crystals-induced autophagy by targeting Akt/mTOR pathway.

In conclusion, our study demonstrated that Tau pretreatment alleviated CaOx crystals-induced oxidative injury in HK-2 cells and kidney tissues. We further showed that Tau suppressed ROS-dependent autophagy via activating Akt/mTOR signaling pathway both *in vitro* and *in vivo*. These results suggest the potential use of Tau for the management of CaOx nephrolithiasis.

## MATERIALS AND METHODS

### Reagents

CaOx (455997), Tau (T0625), 4’,6-diamidino-2-phenylindole (D8417), ethyl pyruvate (E47808), 2′,7′-dichlorofluorescin diacetate (D6883) were ordered from Sigma-Aldrich. MK-2206 2HCl (M129684) was ordered from Aladdin. TUNEL assay kit was purchased from Roche (NJ, USA). The Ad-mRFP-GFP-LC3 adenovirus was purchased from Hanbio, Inc. (Shanghai, China). Antibodies against LC3B (2775), p62 (39786), p-Akt (4060), Akt (4685), p-mTOR (5536), mTOR (2983), IL-1β, β-actin (4970) were obtained from Cell Signaling Technology. Antibodies against IL-1β (ab9722) was obtained from Abcam. Rabbit (ZB-2301) and mouse (ZB-2305) HRP conjugated antibodies were ordered from Golden Bridge Biotechnology (Zhongshan, China).

### Cell culture

HK-2 cells were cultured in complete medium (DMEM/F12 with 10% FBS, 2% penicillin, and 2% streptomycin) at 37°C/5% CO_2_. Cells were divided into five groups: control, Tau, CaOx, CaOx + MK2206 and CaOx + Tau. Cells in the control group were maintained in complete medium; Tau group was pretreated with Tau for 2h and then cultured in complete medium; CaOx group was cultured in complete medium containing 4mmol/L CaOx crystals; CaOx + MK2206 group was pretreated with 10μmol/L MK2206 for 3h and then maintained in complete medium containing 4mmol/L CaOx crystals; CaOx + Tau group was pretreated with Tau for 2h and then maintained in complete medium containing 4mmol/L CaOx crystals. After 24h, cells and supernatants were harvested for further analyses.

### Animal model

Male Sprague-Dawley rats (six-week-old, Laboratory of Animal Resources of Guangxi Medical University) were housed under specific pathogen-free conditions and given free access to sterilized food and water. A rat model of CaOx nephrolithiasis was established by adding 0.75% Ethylene glycol (EG) in drinking water. Rats were randomly assigned into five groups (n=8/group): Control (Feed with normal drinking water), Tau (Feed with normal drinking water + Tau 300 mg/kg/d, i.p.), EG (Feeding with drinking water containing 0.75% ethylene glycol), EG + MK2206 (Feeding with drinking water containing 0.75% ethylene glycol + MK2206 90 mg/kg/48h, i.p.) and EG + Tau (Feeding with drinking water containing 0.75% ethylene glycol + Tau 300 mg/kg/d, i.p.). MK-2206 was resuspended in 30% captisol and intraperitoneally injected in rats at a concentration of 90 mg/kg/48h [41]. Tau was dissolved in stroke-physiological saline solution and intraperitoneally injected in rats at a concentration of 300 mg/kg/d [12]. An equal volume of saline solution was injected in control and EG rats. All animals were sacrificed after 4 weeks. Their kidney tissues and blood samples were collected. A portion of the tissue sample was stored in liquid nitrogen and the rest was fixed in 10% neutral formalin buffer. All experiments were approved by the Institutional Animal Care and Use Committee of Guangxi Medical University.

### Cell viability assay

CCK-8 assay was performed to evaluate the cytotoxicity of Tau. HK-2 cells (5 × 10^4^/mL) were plated in 96-well plates and treated with Tau at 0, 18.75, 37.5, 75 150, 300, 600 or 1200 μmol/L for 24 h. Then cells were incubated with 10% CCK-8 diluted in medium for 2 h at 37°C. The absorbance at 450 nm was detected using a microplate reader. The viability of cells was calculated according to the manufacturer’s instructions.

### Western blot

Proteins were extracted from cells and rat kidney tissues using radioimmunoprecipitation assay buffer. The sample aliquots were stored at -80°C. Protein samples were separated by gel electrophoresis and then transferred to polyvinylidene fluoride membranes. After blocking with 10% BSA, membranes were incubated with designated primary antibodies at 4°C overnight. After three washes, blots were incubated with secondary antibody at room temperature for 60 min and analyzed using Odyssey Fc Imaging System.

### In situ TUNEL fluorescence staining assay

Paraffin-embedded tissue sections were dewaxed, hydrated and permeated for treatment. Slides of cell smears were prepared, fixed with 4% paraformaldehyde, and treated by Triton X-100 (0.3%) for 5 min. A volume of 50 μL TUNEL test solution (45 μL TRITC-dUTP Labeling Mix + 5 μL 10× TdT Enzyme) was added to each sample. Slides were incubated in a wet box at 37 °C in the dark for 60 minutes. After washing with PBS, the nuclei were stained with DAPI. Slides were analyzed under a fluorescence microscope with excitation and emission wavelengths of 546 nm and 570 nm, respectively (red fluorescence).

### Transmission electron microscope

Cell and tissue samples fixed with 2.5% glutaraldehyde were subjected to dehydration, embedding, and solidification to prepare ultrathin slices (50-60 nm). The images were captured using an electron microscope (HITACHI, Tokyo, Japan).

### Enzyme linked-immunosorbent assay (ELISA)

The content of IL-1β in cell-free supernatants was measured using an ELISA kit (Cusabio Biotech, Wuhan, China).

### Adenoviral infection

Briely, HK-2 cells were transduced with adenovirus harboring fluorescent mRFP-GFP-LC3 for 24 h, and subjected to further study. The autophagic flux was evaluated as previously described [9].

### Measurement of intracellular ROS level

ROS detection kit (Solarbio, China) was used to evaluate ROS generation. Cells were incubated with F12 medium containing DCFH-DA solution (10 μM) at 37 °C for 20 min in the dark. After two rinses with PBS, excess DCFH-DA was removed. The images were captured using a fluorescence microscope. The intensity of DCF fluorescence was assessed using a fluorescence spectrophotometer.

### LDH release assay

Cell culture supernatants were collected after model establishment. The concentration of LDH in the supernatants was examined using an LDH Cytotoxicity Assay Kit (Jiancheng Bioengineering Institute, Nanjing, China). The absorbance at 450 nm was read using a microplate reader (Thermo Fisher Scientific).

### Measurement of MDA and SOD levels

Cells were collected after model establishment. The concentrations of MDA and SOD in cells or tissue samples were assessed using MDA and SOD Assay Kit (Jiancheng Bioengineering Institute, Nanjing, China) following the manufacturer’s protocol.

### Mitochondrial membrane potential (Δψm) detection

A JC-1 staining kit (Solarbio, China) was used to detect mitochondrial membrane potential. Cells were collected and resuspended in culture medium containing 50% JC-1 staining fluid. After 20-min incubation with JC-1 staining fluid at 5% CO_2_/37°C, cell were washed twice with JC-1 staining buffer. The green fluorescence (excitation 515 nm; emission 529 nm) and red fluorescence (excitation 585 nm; emission 590 nm) were measured using a fluorescence microplate reader.

### Immunohistochemical staining

Rat kidney samples were fixed with 4% paraformaldehyde, embedded in paraffin, and sectioned into 3μm thick slices. After dewaxing and dehydrating, sodium citrate at 0.01 mol/L was used to retrieve antigen by high pressure. Hydrogen peroxide (3%) was used to remove endogenous peroxide. After blocking with goat serum, slides were stained with primary antibody overnight at 4 °C, followed by the incubation with secondary antibody at room temperature for half an hour. Then slides were stained with 3,3-diaminoaniline, counterstained with hematoxylin, and observed under a microscope (Olympus C-5050, Japan).

### Von Kossa staining

Prepared tissue sections (5 μm) were dewaxed, dehydrated, added with 1% silver nitrate solution, and irradiated under sunlight for 30 minutes. Then silver nitrate solution was discarded and samples were incubated with 5% sodium thiosulfate solution for 1 min. After stained with basic fuchsin for 10 s, samples were transparentized, dehydrated, and sealed with neutral resin.

### Statistical analysis

All data were shown as mean ± SD from three experiments and analyzed using SPSS (20.0). Significant difference among three or more groups was determined by one-way analysis of variance. Significant difference between two groups was evaluated using Student’s t-test. *P* < 0.05 was considered statistically significant. ^*^ indicates *P* < 0.05 vs. control group. ^#^ indicates *P* < 0.05 vs. CaOx or EG group.
