# Correction for: Taurine suppresses ROS-dependent autophagy via activating Akt/mTOR signaling pathway in calcium oxalate crystals-induced renal tubular epithelial cell injury

**DOI:** 10.18632/aging.202587

**Published:** 2021-01-27

**Authors:** Yan Sun, Shiting Dai, Jin Tao, Yunlong Li, Ziqi He, Quan Liu, Jiawen Zhao, Yaoliang Deng, Juening Kang, Xuepei Zhang, Sixing Yang, Yunlong Liu

**Affiliations:** 1 Department of Urology, The First Affiliated Hospital of Zhengzhou University, Zhengzhou, China; 2 Department of Urology, Renmin Hospital of Wuhan University, Wuhan, China; 3 Department of Urology, The First Affiliated Hospital of Guangxi Medical University, Nanning, China

**Keywords:** correction

Original article: Aging. 2020; 12:17353–17366.  . https://doi.org/10.18632/aging.103730

**This article has been corrected:** The authors requested to remove National Natural Science Foundation of China (No. 81760127) from the list of funding organizations because this work was mainly funded by Joint Construction Project between Medical Science and Technology Research Project of Henan Province (No. LHGJ20190179). The name of Dr. Deng Yaoliang has been removed from the list of authors since as a holder of above-mentioned fund, Dr. Deng Yaoliang believes that his input to this article was not substantially enough for the authorship. This correction has not changed the interpretation of the original conclusions of this publication.

The corrected list of authors and Funding information are provided below:

Yan Sun^1,*^, Shiting Dai^1,*^, Jin Tao^1^, Yunlong Li^1^, Ziqi He^2^, Quan Liu^3^, Jiawen Zhao^3^, Juening Kang^3^, Xuepei Zhang^1^, Sixing Yang^2^, Yunlong Liu^1^

