# Correction 2 for: Taurine suppresses ROS-dependent autophagy via activating Akt/mTOR signaling pathway in calcium oxalate crystals-induced renal tubular epithelial cell injury

**DOI:** 10.18632/aging.206246

**Published:** 2025-04-30

**Authors:** Yan Sun, Shiting Dai, Jin Tao, Yunlong Li, Ziqi He, Quan Liu, Jiawen Zhao, Yaoliang Deng, Juening Kang, Xuepei Zhang, Sixing Yang, Yunlong Liu

**Affiliations:** 1 Department of Urology, The First Affiliated Hospital of Zhengzhou University, Zhengzhou, China; 2 Department of Urology, Renmin Hospital of Wuhan University, Wuhan, China; 3 Department of Urology, The First Affiliated Hospital of Guangxi Medical University, Nanning, China

**Keywords:** autophagy, calcium oxalate crystals, reactive oxygen species, renal tubular epithelial cells, taurine

**This article has been corrected:** The authors recently discovered an overlap between two TUNEL staining images in **Figure 2D**. The authors inadvertently assembled incorrect images in the revised manuscript. The TUNEL staining image intended for the EG+Tau group was mistakenly used for the EG group. The authors replaced incorrect image with the original image from the EG group staining and stated that this correction does not affect any of the results or conclusions of the paper. The authors apologize for any inconvenience this error may have caused.

The corrected version of **Figure 2D** is provided below.

**Figure 2:**
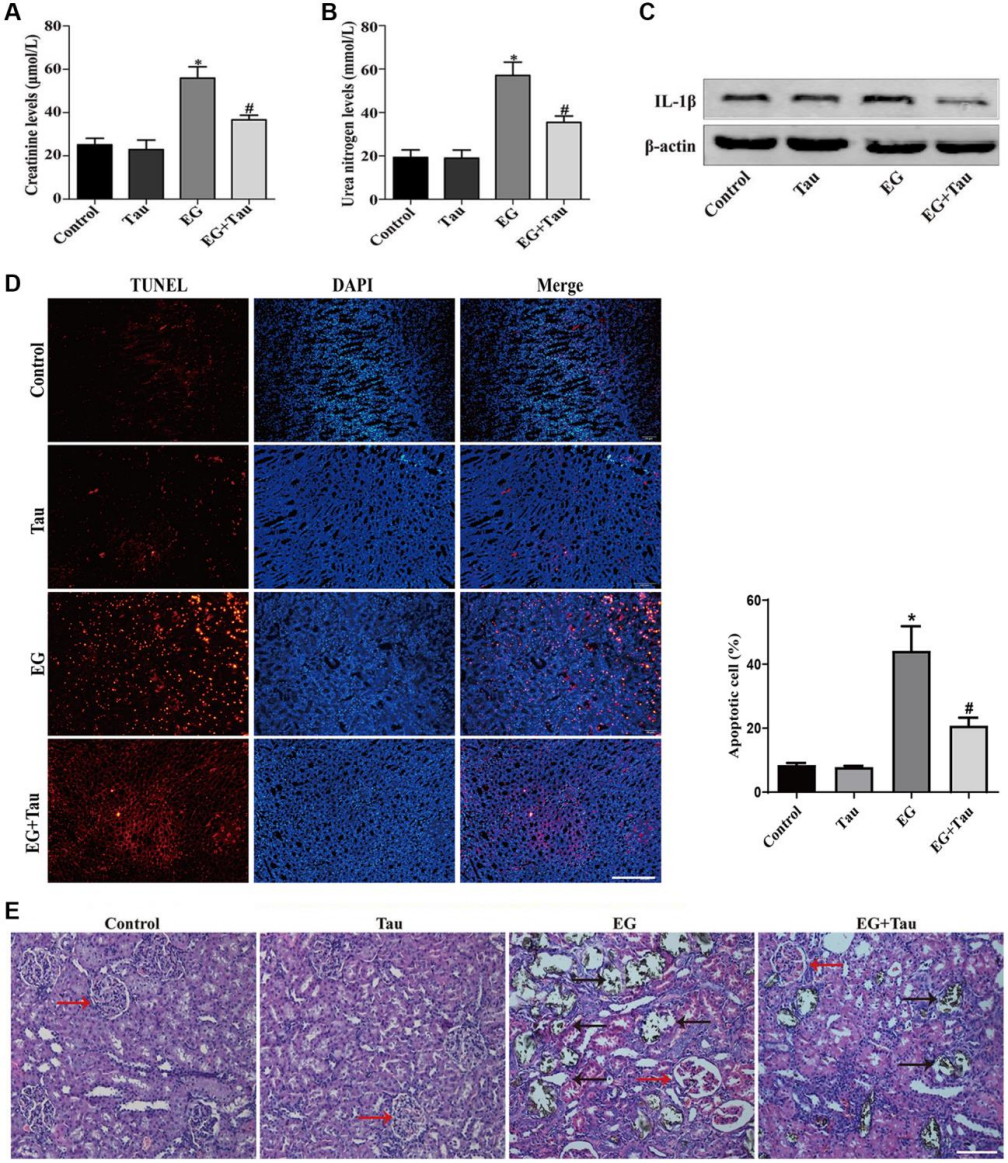
**Tau attenuates EG-induced renal damage and crystal deposition in rat kidneys.** (**A**) Effect of Tau on the serum expression of creatinine after EG-induced renal injury. (**B**) Effect of Tau on urea nitrogen following EG-induced renal injury. (**C**) Representative immunoblot and quantification analysis of IL-1β expression. (**D**) Renal tissue apoptosis was assessed by TUNEL staining; scale bar: 200 µm. (**E**) Kidney injury and crystal deposition were determined using Von Kossa-staining. Red and black arrows indicate glomerulus and crystal deposition, respectively; scale bar: 200 µm. Data are presented as the mean ± SD (*n* = 3). ^*^P < 0.05 versus the control group, ^#^*P* < 0.05 versus the EG group.

